# Differential molecular response of monodehydroascorbate reductase and glutathione reductase by nitration and *S*-nitrosylation

**DOI:** 10.1093/jxb/erv306

**Published:** 2015-06-25

**Authors:** Juan C. Begara-Morales, Beatriz Sánchez-Calvo, Mounira Chaki, Capilla Mata-Pérez, Raquel Valderrama, María N. Padilla, Javier López-Jaramillo, Francisco Luque, Francisco J. Corpas, Juan B. Barroso

**Affiliations:** ^1^Group of Biochemistry and Cell Signaling in Nitric Oxide, Biochemistry and Molecular Biology Division, Department of Experimental Biology, Faculty of Experimental Sciences, University of Jaén, Campus ‘Las Lagunillas’, E-23071 Jaén, Spain; ^2^Institute of Biotechnology, University of Granada, E-18071 Granada, Spain; ^3^Center for Advanced Studies in Olives and Olive Oil, University of Jaén, Campus ‘Las Lagunillas’, E-23071 Jaén, Spain; ^4^Group of Antioxidants, Free Radicals and Nitric Oxide in Biotechnology, Food and Agriculture, Department of Biochemistry, Cell and Molecular Biology of Plants, Estación Experimental del Zaidín, CSIC, Apartado 419, E-18080 Granada, Spain

**Keywords:** Glutathione reductase, monodehydroascorbate reductase, nitration, nitric oxide, peroxynitrite, reactive nitrogen species, salinity, *S*-nitrosylation, *S*-nitrosoglutathione.

## Abstract

The ascorbate–glutathione cycle is a metabolic pathway that detoxifies hydrogen peroxide and involves enzymatic and non-enzymatic antioxidants. Proteomic studies have shown that some enzymes in this cycle such as ascorbate peroxidase (APX), monodehydroascorbate reductase (MDAR), and glutathione reductase (GR) are potential targets for post-translational modifications (PMTs) mediated by nitric oxide-derived molecules. Using purified recombinant pea peroxisomal MDAR and cytosolic and chloroplastic GR enzymes produced in *Escherichia coli*, the effects of peroxynitrite (ONOO^–^) and *S*-nitrosoglutathione (GSNO) which are known to mediate protein nitration and *S*-nitrosylation processes, respectively, were analysed. Although ONOO^–^ and GSNO inhibit peroxisomal MDAR activity, chloroplastic and cytosolic GR were not affected by these molecules. Mass spectrometric analysis of the nitrated MDAR revealed that Tyr213, Try292, and Tyr345 were exclusively nitrated to 3-nitrotyrosine by ONOO^–^. The location of these residues in the structure of pea peroxisomal MDAR reveals that Tyr345 is found at 3.3 Å of His313 which is involved in the NADP-binding site. Site-directed mutagenesis confirmed Tyr345 as the primary site of nitration responsible for the inhibition of MDAR activity by ONOO^–^. These results provide new insights into the molecular regulation of MDAR which is deactivated by nitration and *S*-nitrosylation. However, GR was not affected by ONOO^–^ or GSNO, suggesting the existence of a mechanism to conserve redox status by maintaining the level of reduced GSH. Under a nitro-oxidative stress induced by salinity (150mM NaCl), MDAR expression (mRNA, protein, and enzyme activity levels) was increased, probably to compensate the inhibitory effects of *S*-nitrosylation and nitration on the enzyme. The present data show the modulation of the antioxidative response of key enzymes in the ascorbate–glutathione cycle by nitric oxide (NO)-PTMs, thus indicating the close involvement of NO and reactive oxygen species metabolism in antioxidant defence against nitro-oxidative stress situations in plants.

## Highlight

Nitration and *S*-nitrosylation, two post-translational modifications (PTMs) mediated by nitric oxide, differentially regulate MDAR and GR activities which are key components of the ascorbate–glutathione cycle.

## Introduction

The ascorbate–glutathione cycle is composed of monodehydroascorbate reductase (MDAR), glutathione reductase (GR), ascorbate peroxidase (APX), and dehydroascorbate reductase (DHAR), as well as the antioxidant metabolites ascorbate and glutathione and the reductive coenzyme NADPH. This metabolic pathway is essential for the detoxification and regulation of the cellular level of hydrogen peroxide (H_2_O_2_) in plant cells (Hossain and Asada, 1985; Asada, 1992; Noctor and Foyer, 1998; Shigeoka *et al.*, 2002). Thus, H_2_O_2_ is reduced to water by APX using ascorbate as the electron donor. The oxidized ascorbate (monodehydroascorbate or dehydroascorbate) is then regenerated by MDAR and DHAR using reduced glutathione (GSH) with the concomitant generation of oxidized glutathione (GSSG). Finally, GSSG is reduced by GR with the aid of NADPH as the electron donor. At the subcellular level, these enzymes have been demonstrated to be located in different cellular compartments including the cytosol, chloroplasts, peroxisomes, and mitochondria (Foyer and Halliwell, 1976; Groden and Beck, 1979; Jiménez *et al.*, 1998; Asada, 2006; Palma *et al.*, 2006; Reumann and Corpas, 2010).

Nitric oxide (NO) belongs to a family of related molecules known as reactive nitrogen species (RNS). Although the specific source of NO in plants is still under debate (see Corpas *et al.*, 2009), there is no doubt that plants have an endogenous NO source(s). *S*-Nitrosoglutathione (GSNO) is formed by the *S*-nitrosylation reaction of NO with GSH, and it has a significant physiological relevance because GSNO is considered to function as a mobile reservoir of NO bioactivity. On the other hand, the reaction of NO with superoxide radicals (O_2_·^–^) generates a powerful oxidant, designated peroxynitrite (ONOO^–^). Furthermore, these NO-derived molecules can mediate several post-translational modifications (PTMs) such as nitration and *S*-nitrosylation. Protein tyrosine nitration involves the addition of a nitro group (-NO_2_) to one of the two equivalent ortho-carbons of the tyrosine residue aromatic ring. Protein nitration is affected by various features such as the protein quaternary structure, the environment in which the protein is located, and the nitration mechanism. Consequently, these covalent changes may result in effects such as loss or gain of protein function or no change in function (Souza *et al*., 2008; Radi, 2013). On the other hand, *S*-nitrosylation involves the binding of an NO group to a protein cysteine residue and is also able to change the function of many proteins (Astier *et al.*, 2012). Proteomic studies have identified potential plant protein candidates for nitration and *S*-nitrosylation which belong to diverse functional categories such as redox-related, stress-related, metabolic, and signalling/regulating proteins (Lindermayr *et al.*, 2006; Corpas *et al.*, 2015). These analyses have identified some enzymatic components of the ascorbate–glutathione cycle as potential PTM targets mediated by NO-derived molecules. However, little is known about the specific impact of these NO-related PTMs on the activity and structure of particular proteins involved in antioxidative systems (Holtgrefe *et al.*, 2008; Chaki *et al.*, 2011; Astier *et al.*, 2012; Begara-Morales *et al.*, 2013*a*, 2014). In previous studies, in order to understand the fine-tuned regulation of this key antioxidant system by NO, the effect of NO-related PTMs on the activity of cytosolic APX was analysed. Thus, APX was identified as a target of *S*-nitrosylation in *Arabidopsis* plants (Fares *et al.*, 2011; Keyster *et al.*, 2011), and pea cytosolic APX has very recently been demonstrated to have a dual mechanism of regulation mediated by NO-derived molecules; while the nitration of Tyr235 provoked a deactivation of APX activity, *S*-nitrosylation at Cys32 activated this activity (Begara-Morales *et al.*, 2014). On the other hand, a nitroproteomic study of sunflower hypocotyls identified GR as a target for tyrosine nitration (Chaki *et al.*, 2009) and for *S*-nitrosylation in rice (Lin *et al.*, 2012). Additionally, MDAR has also been identified as a potential candidate for both *S*-nitrosylation and nitration in citrus plants (Tanou *et al.*, 2012) and for *S*-nitrosylation in rice (Lin *et al.*, 2012) and *Arabidopsis* (Hu *et al.*, 2015). However, to the authors’ knowledge, no information is available on the effect of any PTMs mediated by NO-derived molecules on the molecular function of MDAR or GR.

Thus, the aim of the present study is to gain a more in-depth understanding of the regulation of the antioxidative ascorbate–glutathione cycle by NO-PTMs. Pea (*Pisum sativum*) was selected as the model plant for several reasons: (i) previous studies have allowed significant information on NO metabolism to be obtained (Barroso *et al.*, 2006; Corpas *et al.*, 2008, 2013*b*); (ii) the isolation and characterization of a full-length genomic clone encoding the pea MDAR has been reported; and (iii) a previous study on pea APX showed dual regulation by *S*-nitrosylation and nitration (Begara-Morales *et al.*, 2014). On the other hand, both chloroplastic and cytosolic isoforms of GR (Edwards *et al.*, 1990; Stevens *et al.*, 1997; Gill *et al.*, 2013; Wu *et al.*, 2013) were used to obtain the most complete information about this enzyme. Consequently, using *in vitro* approaches, the potential effect of NO-derived molecules such as ONOO^–^ and GSNO, which trigger nitration and *S*-nitrosylation, respectively, on the molecular function of two enzymes (peroxisomal MDAR and cytosolic and chloroplastic GR) in this cycle was analysed.

As a result, peroxisomal MDAR was deactivated by both nitration and *S*-nitrosylation, which compromise the cycle’s antioxidant capacity. However, cytosolic and chloroplastic GR were unaffected by any of these NO-PTMs in an attempt to maintain the levels of GSH and the cellular redox state.

## Materials and methods

### Plant material and growth conditions

Pea (*Pisum sativum* L., cv. Lincoln) seeds were obtained from Royal Sluis (Enkhuizen, The Netherlands). Seeds were surface sterilized with 3% (v/v) commercial bleach solution for 3min, and then were washed with distilled water, and germinated in vermiculite for 3–4 d under the following growth chamber conditions: 24 ºC/18 °C (day/night), 80% relative humidity, a 16h photoperiod, and a light intensity of 190 μE m^–2^ s^–1^. Healthy and vigorous seedlings were selected and grown in nutrient solutions (Corpas *et al.*, 1993). After 14 d, plants were transplanted to similar media supplemented with 150mM NaCl and were grown for 4 d (Begara-Morales *et al.*, 2014).

### Expression and purification of pea MDAR and GR

The cDNAs encoding mature pea peroxisomal *MDAR* (AY662655.1) and cytosolic (X98274.1) and chloroplastic (X60373.1) *GR* were amplified by PCR from total pea leaf RNA using the Fast Start High Fidelity polymerase (Roche) and the specific primer sets: 5′- GGATCCGATGGTGCATTCGTTCAAGTATATC-3′ (forward) and 5′-GCTCGAGTATTAAATTTTACTTGCAAA AGAAAGG-3′ (reverse) for *MDAR*; 5′-AGGATCCAATGAA CCAAGCAATGGCTACTC-3′ (forward) and 5′-CTCGAGTCTTA AGATCCAGCCACAGCTTTTG-3′ (reverse) for chloroplastic *GR*; and 5′-GGATCCGATGGCTAGAAAGATGCTTAACG-3′ (forward) and 5′-CTCGAGTTTTACAATTTGTCTT TGGCT TCAC-3′ (reverse) for cytosolic *GR*. The PCR products (1316bp for *MDAR*, 1671bp for chloroplastic *GR*, and 1510bp for cytosolic *GR*) were cloned into the pGEM-T Easy Vector (Promega). The positive clones were confirmed by sequencing and then subcloned following prior digestion with *Bam*HI and *Xho*I into the pALEXb vector.

Recombinant proteins carrying an N-terminal choline-binding domain were produced using *Escherichia coli* strain BIVU0811, which were routinely cultured overnight at 37 ºC in LB with kanamycin (25mg l^–1^) and ampicillin (100mg l^–1^). Gene expression was induced by the addition of 1mM salicylate and 10mM 3-methyl benzoate in a 250ml culture grown at 20 ºC overnight in order to produce a higher proportion of soluble protein. Cells were harvested by centrifugation and re-suspended in 20ml of phosphate-buffered saline (PBS; pH 7.0) containing 25U ml^–1^ DNAse I, 10mM MgCl_2_, and commercial protease inhibitor (Complete, Roche). Cells were lysed with a Niro Soavi NS1001L Panda High-Pressure homogenizer at a pressure of 800–900 bar. The cell lysate was then centrifuged at 10 000 *g* at 4 °C for 15min, and the supernatant was used for the purification of recombinant proteins with the aid of a 1ml LYTRAP column (Biomedal). The column was washed with 20ml of 20mM K phosphate buffer (pH 7.0) containing 300mM NaCl and 5mM choline. The protein was eluted in 1ml fractions using a discontinuous gradient of choline prepared in the same buffer with 100mM NaCl and 20mM choline (fraction E1), 50mM choline (E2), 75mM choline (E3), 100mM choline (E4), 150mM choline (E5), 200mM choline (E6), 250mM choline (E7), and 500mM choline (E8). The samples were analysed by 10% SDS–PAGE and stained with Coomassie blue dye. Supplementaryy Fig. S1 available at *JXB* online shows the SDS–PAGE analysis of the purification of recombinant peroxisomal MDAR (Supplementary Fig. S1A) and chloroplastic and cytosolic GR (Supplementary Fig. S1B, C).

### MDAR and GR activity assays: treatment with a peroxynitrite donor (SIN-1) and a nitric oxide donor (GSNO)

MDAR (EC 1.6.5.4) activity was determined spectrophotometrically by measuring the reduction of absorbance at 340nm according to the technique described by Hossain *et al.* (1984) with some modifications. The 1.0ml assay mixtures contained 50mM TRIS-HCl (pH 7.8), 0.2mM NADH, 1mM ascorbate, and sample. The reaction was initiated by adding 0.2U of ascorbate oxidase (EC 1.10.3.3 from *Cucurbita*; Sigma-Aldrich, St. Louis, MO. USA), and the decrease in *A*_340_ due to NADH oxidation was monitored. One milliunit of activity was defined as the amount of enzyme required to oxidize 1 nmol NADH min^–1^ at 25 ºC. GR (EC 1.6.4.2) activity was assayed by monitoring NADPH oxidation coupled with the reduction in GSH (Edwards *et al.*, 1990). The reaction rate was corrected for the slight non-enzymatic oxidation of NADPH by glutathione disulphide (GSSG).

The molecule SIN-1 (3-morpholinosydnonimine) has been shown to generate peroxynitrite (ONOO^–^), a protein-nitrating compound (Daiber *et al.*, 2004). Recombinant proteins were therefore incubated at 37 °C for 1h with increasing concentrations (0–5mM) of SIN-1 (Calbiochem) freshly made up before use (Begara-Morales *et al.*, 2014). For treatments with GSNO (NO donor), recombinant proteins were incubated at room temperature for 30min with 0.5mM and 2mM GSNO (Begara-Morales *et al.*, 2014). As a control, proteins were also incubated with 0.5mM and 2mM GSH. The protein concentration was determined with the aid of the Bio-Rad protein assay using bovine serum albumin (BSA) as standard.

### Identification of nitrated tyrosine residues in recombinant pea MDAR using mass spectrometric techniques

Purified recombinant pea MDAR was processed according to a protocol involving reduction with dithiothreitol (DTT), derivatization with iodoacetamide (IAA), and enzymatic digestion with trypsin (37 ºC, 8h). The sample was purified using solid-phase extraction cartridges to eliminate choline interference. The resulting peptide mixture was analysed using a MALDI-TOF/TOF (matrix-assisted laser desorption ionization-time of flight/time of flight) mass spectrometer (4800, AB Sciex) to evaluate the quality of the sample. MALDI-TOF spectra were interpreted using a peptide mass fingerprinting (PMF) database search (Protein Prospector program). The database used for identification was UniProt (release 2011_02). The sample was then analysed by liquid chromatography–tandem mass spectometry (LC-MS/MS) using a Velos-LTQ mass spectrometer equipped with a micro-ESI ion source (ThermoFisher, San Jose, CA, USA). The sample was evaporated to dryness and diluted up to 40 μl with water containing 5% methanol and 1% formic acid. The sample was then loaded in a chromatographic system consisting of a C18 pre-concentration cartridge (Agilent Technologies, Santa Clara, CA, USA) connected to a 10cm long, 150 μm id Vydac C18 column (Vydac, IL, USA). Separation was carried out at 1 μl min^–1^ with a 3–40% acetonitrile gradient for 30min (solvent A, 0.1% formic acid; solvent B, acetonitrile with 0.1% formic acid). The high-performance liquid chromatography (HPLC) system contained an Agilent 1200 capillary pump, a binary pump, a thermostated microinjector, and a micro switch valve.

The Velos-LTQ instrument was operated in positive ion mode with a spray voltage of 2kV. The scan range of each full MS scan was *m/z* 400–2000. After each MS scan, a collection of targeted MS/MS spectra was obtained in order to identify both the unmodified and nitrated form of the predicted tyrosine-containing peptides. The parent mass list of the targeted scan was selected to ensure maximum coverage of the tyrosine-containing tryptic peptides for MDAR. The list of targeted *m/z* values was obtained after *in silico* digestion of the proteins using nitrated tyrosine as a dynamic modification. The resulting list of predicted peptides (in both nitrated and unmodified form) was filtered to exclude all peptides not containing tyrosine residues.

The MS/MS spectra were searched using Proteome Discoverer software (ThermoFisher) on the basis of the following parameters: peptide mass tolerance 2Da, fragment tolerance 0.8Da, enzyme set as trypsin, and no missed cleavages. The dynamic modifications were cysteine carbamidomethylation (+57Da), methionine oxidation (+16Da), and tyrosine nitration (+45). The searches were carried out using a database containing all the peptides listed in Table 1. Identifications were filtered with XCorr >3, P(pep) <15%. The MS/MS spectra of the nitrated tyrosines were manually validated by comparing the spectra obtained for the unmodified peptide and the nitrated peptide.

**Table 1.** List of pea MDAR peptides scanned and identified by LC-MS/MS

Peptides identified^*a*^	Peptides scanned	Length (no. of amino acids)	*M*_r_ (Da)		No. of tyrosine residues
			Not nitrated	Nitrated	
AKPAVEDVNQLAEEGLSFASK		21	2203		0
AVVVGGGYIGLELSAVLK	AVVVGGGYIGLELSAVLK	18	1745		1
AYLFPESPAR	AYLFPESPAR	10	1150		1
EAVAPYERPALSK	EAVAPYERPALSK	13	1431		1
	FGTYWIK	7	914		1
GIQLYLSTEIVSADLAAK	GIQLYLSTEIVSADLAAK	18	1892		1
LFTSEIAAFYEGYYANK	LFTSEIAAFYEGYYANK	17	1987	2032	3
	LLPEWYSEK	9	1164		1
	LNDLDVTMVYPEPWCMPR	18	2180		1
LPGFHTCVGSGGER		14	1417		0
	NIFYLR	6	825		1
SANGEHFDYQTLVIATGSAVIR	SANGEHFDYQTLVIATGSAVIR	22	2350		1
SFDLSWQFYGDNVGETVLFGDNDPASSKPK	SFDLSWQFYGDNVGETVLFGDNDPASSKPK	30	3322		1
SVEEYDYLPYFYSR	SVEEYDYLPYFYSR	14	1831	1876	4
TSVPDVYAVGDVATFPLK	TSVPDVYAVGDVATFPLK	18	1879	1924	1
YILIGGGVSAGYAAR	YILIGGGVSAGYAAR	15	1468		2

Some peptides detected do not contain tyrosines. These peptides were not included in the targeted MS/MS detection. They were detected and identified as their molecular weight coincides with that of predicted peptides.

### Site-directed mutagenesis

Conversion of the tyrosine codon (TAT) to phenylalanine (TTT) in the pea cDNA of peroxisomal MDAR (accession no, AY662655) was accomplished by oligonucleotide-directed mutagenesis. The template for PCR mutagenesis was also the pALEXb expression vector carrying the entire *P. sativum* pMDAR I gene obtained as described above. The site-directed mutant of MDAR Tyr345Phe was obtained by using the following primers: 5′-CTTTGATCTTTTCCAATCCAC-3′ (MDAR Tyr345Phe forward) and 5′-GTGGATTGGAAAAGATCAAAG-3′ (MDAR Tyr1345Phe reverse) where the mutated nucleotides are underlined. The mutation was introduced using the QuikChange kit following the manufacturer’s protocol. The mutant plasmid encoding nucleotide sequence was confirmed by DNA sequencing. The plasmids containing the mutation were transformed into XL1-Blue supercompetent cells and stored at –80 °C in 85% glycerol. The expression and purification of the recombinant mutant MDAR protein were obtained as previously described for the wild-type MDAR protein.

### *In silico* analysis of MDAR

The three-dimensional structure of pea peroxisomal MDAR was modelled at the Geno3D server (Combet *et al.*, 2002) using as template the structures of the putidaredoxin reductase from *Pseudomonas putida* (PDB code access 1q1w and 1q1r) (Sevrioukova *et al.*, 2004) and the ferredoxin reductase from *Rhodopseudomonas palustris* (PDB code access 3fg2) (Xu *et al*., 2009) with an identity of 26.8% and 30.1%, respectively. The analysis of the quality of the models was carried out at the Structural Analysis and Verification Server (SAVES) in terms of atomic non-local environment assessment (ANOLEA) (Melo and Feytmans, 1998), three-dimensional profiles (Verify3D) (Eisenberg *et al.*, 1997), and Procheck (Laskowski *et al.*, 1993). The co-ordinates of FAD and NAD were calculated by superposition of the model on those of the X-ray structure 2YVG, which shares 30% identity with the primary structure of the pea MDAR and 1.55 Å rms (backbone atoms) with the pea MDAR model.

Docking of the model of pea MDAR with GSH was carried out at the SwissDock sever (Grosdidier *et al*., 2011) in accurate mode and without defining the region of interest (blind docking) but allowing flexibility for the side chains within 5 Å of any atom of the ligand in its reference binding mode. Analysis of the results was carried out with the help of Swiss PdbViewer (Guex and Peitsch, 1997) and UCSF Chimera (Pettersen *et al*., 2004)

Molecular evolution studies were carried out at the Evolutionary Trace server (Mihalek *et al.*, 2004) using the model of the tertiary structure as input. The phylogenetic significance and evolutionary conservation were explored by BLASTP searches (Altschul *et al.*, 1997) on the subsection Viridiplantae of UniProtKB release 2013_05 (UniProt Consortium, 2011). The phosphorylation score was computed at the NetPhos 2.0 Server (Blom *et al.*, 1999), and the solvent-accessible area by DSSP (Kabsch and Sande, 1983). The estimation of the p*K*a and the analysis of the interactions was carried out with Propka 3.1 (Olsson *et al*., 2011). The algorithm was originally described by Li *et al*. (2005) as a very fast empirical method for structure-based p*K*a prediction that relies on the estimation of desolvation effects and intraprotein interactions to account for the variation in the standard p*K*a of ionizable groups.

### Biotin switch method

For *in vitro S*-nitrosylation, peroxisomal MDAR and chloroplastic and cytosolic GR were incubated with GSNO as the NO donor for 30min at room temperature. *S*-Nitrosylated recombinant proteins were subjected to the biotin switch method as described by Begara-Morales *et al.* (2014). The non-nitrosylated free cysteine residue was blocked by incubation with 30mM methyl methane thionsulphonate and 2.5% SDS at 50 °C for 20min with frequent vortexing. Residual methyl methane thionsulphonate was removed by precipitation with 2 vols of –20 °C acetone, and the proteins were resuspended in 0.1ml of HENS buffer (25mM HEPES pH 7.7 buffer containing 1mM EDTA, 0.1mM neocuproine, and 1% SDS) per milligram of protein. Biotinylation was obtained by adding 1mM *N*-[6-(biotinamido) hexyl]-3′-(2′-pyridyldithio) propionamide (biotin-HPDP) and 0.1mM ascorbate, and incubating at room temperature for 1h. The proteins were then precipitated with 2 vols of –20 ºC acetone. Biotin-labelled proteins were separated by non-reducing 10% SDS–PAGE and then transferred onto polyvinylidene difluoride (PVDF) membranes (Immobilon P, Millipore, Bedford, MA, USA) using a semi-dry transfer system (Bio-Rad Laboratories). PVDF membranes were blocked using TRIS-buffered saline (TBS)+1% BSA. The blots were incubated with anti-biotin antibody at a dilution of 1:20 000 for 1h, and the immunoreactive bands were detected using a photographic film (Hyperfilm, Amersham Pharmacia Biotech) with an enhanced chemiluminescence kit (ECL-PLUS, Amersham Pharmacia Biotech).

### Purification of biotinylated proteins and immunodetection of MDAR

Purification of biotinylated proteins from control and NaCl-treated pea plant leaves was carried out as described by Begara-Morales *et al.* (2014). Briefly, biotinylated proteins and 30 μl of neutravidin agarose 50% (w/v) slurry (high capacity neutravidin agarose resin, Thermo Scientific) per milligram of protein were equilibrated with a neutralization buffer [20mM HEPES pH 7.7 containing 100mM NaCl, 1mM EDTA, and 0.5% (v/v) Triton X-100]. Proteins were added to the neutravidin agarose matrix and were incubated 1h at room temperature with gentle shaking. The matrix with bound proteins was washed several times with washing buffer [20mM HEPES pH 7.7 containing 600mM NaCl, 1mM EDTA, and 0.5% (v/v) Triton X-100] and was transferred to an empty column. Finally, biotinylated proteins were eluted after incubation for 30min with elution buffer (20mM HEPES pH 7.7 containing 0.1M NaCl, 1mM EDTA, and 100mM β-mercaptoethanol) at room temperature. Purified biotinylated proteins were separated by 12% SDS–PAGE and transferred to PVDF membranes as described above.

For MDAR immunodetection, the membrane was incubated with a rabbit polyclonal antibody against cucumber MDAR (Sano and Asada, 1994) diluted 1:3000. Immunoreactive bands were detected using a photographic film (Hyperfilm, Amersham Pharmacia Biotech) with an enhanced chemiluminescence kit (ECL-PLUS, Amersham Pharmacia Biotech).

### Real-time quantitative RT–PCR

Real-time quantitative reverse transcription–PCR (RT–PCR) was performed in 20 μl of reaction mixture, composed of 1 μl of cDNA, master mix IQ™ SYBR Green Supermix (Bio-Rad Laboratories, Hercules, CA, USA), and 10 pmol gene-specific forward and reverse oligonu cleotides (5′-AGAAGAATGCGAAAGCTGTGGTTGTTGGAG-3′ and 5′-TGCTTCCAGGACCCTACCATCCTTTAGTTT-3′, respec tively) for pea *MDAR* using a iCycler iQ system (Bio-Rad). Amplifications were performed under the following conditions: initial polymerase activation, 95 ºC for 5min; then 35 cycles of 30 s at 95ºC, 30 s at 62.2 ºC, and 1min at 72ºC; with a final extension at 72ºC for 7min. An internal control of *18S rRNA* was used for the normalization with the following forward and reverse oligonucleotides: 5′-GTGCAACAAACCCCGACTTTTGAAGGATG-3′ and 5′-GTGGTAGCCGTTTCTCAGGCTCCCTCTC-3′.

## Results

### Expression and purification of recombinant pea MDAR and GR proteins: effect of peroxynitrite

In order to increase the knowledge of the regulatory mechanism of the ascorbate–glutathione cycle involved in the decomposition of H_2_O_2_, the recombinant proteins of two of its components, the MDAR and GR isoforms, were obtained by sequencing the pea clones and overexpression in *E. coli* (see the Materials and methods). Supplementary Fig. S1 at *JXB* online shows the electrophoretic analysis of the different fractions obtained after LYTRAP affinity column chromatography of recombinant MDAR and GR proteins. Recombinant MDAR (Supplementary Fig. 1A) showed a molecular mass of 68.6kDa, which is within the range of the theoretical value predicted for the peroxisomal MDAR protein (47.3kDa) with the Ly-tag (21.3kDa). The fractions E3–E5 with an MDAR activity of 1200 nmol NADH min^–1^ mg^–1^ protein showed an adequate grade of purity for this protein which was used for subsequent experiments. On the other hand, the recombinants chloroplastic and cytosolic GR (Supplementary Fig. 1B, C) showed a molecular mass of ~81kDa and 75kDa, respectively, which is within the range of the theoretical value predicted for both GR proteins (59.7kDa with the Ly-tag 21.3kDa for the chloroplastic isoform, and 53.7kDa with the Ly-tag 21.3kDa for the cytosolic isoform). The fractions E2–E4 with GR activities of 18 μmol NADPH min^–1^ mg^–1^ protein were used for subsequent experiments.

**Fig. 1.**
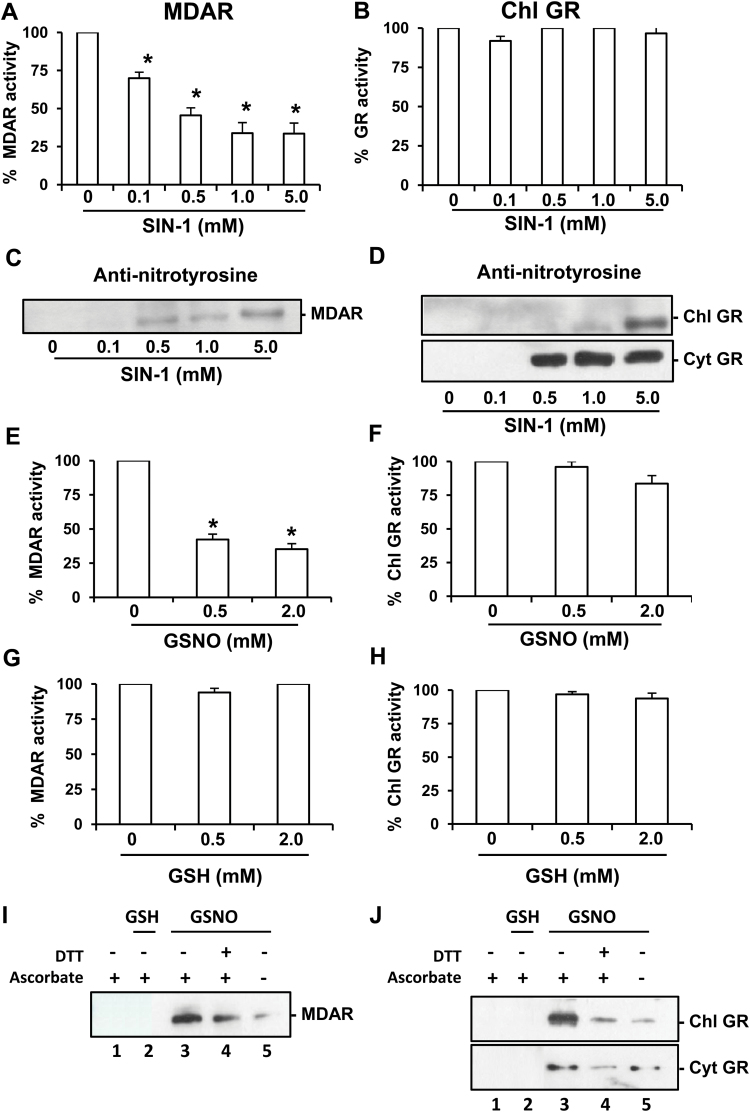
Effect of nitration and *S*-nitrosylation on recombinant monodehydroascorbate reductase (MDAR) and glutathione reductase (GR). Effect of SIN-1 (peroxynitrite donor) on recombinant MDAR (A) and GR (B) activities. Representative immunoblot showing the grade of tyrosine nitration of MDAR (C) and chloroplastic and cytosolic GR (D), treated with different concentrations of SIN-1 and detected with an antibody against 3-nitrotyrosine (dilution 1:2500). A 5 μg aliquot of protein was used per line. Effect of *S*-nitrosoglutathione (GSNO) on recombinant MDAR (E) and chloroplastic GR (F). Effect of glutathione (GSH) on recombinant MDAR (G) and chloroplastic GR (H). Purified MDAR and GR proteins were incubated at different concentrations of SIN-1 at 37 ºC for 60min, GSNO at 25 ºC for 30min, or GSH at 25 ºC for 30min. The specific activity of the recombinant MDAR was 1200 nmol NADH min^–1^ mg^–1^ and for GR proteins it was 18 μmol NADPH min^–1^ mg^–1^. *S*-Nitrosylation of recombinant MDAR (I) and chloroplastic and cytosolic GR (J). A 5 μg aliquot of purified recombinants proteins was treated with 2mM GSH and 2mM GSNO and was subjected to the biotin switch method. Control treatments were carried out with water (lane 1) and 2mM GSH (lane 2). Additionally, recombinants proteins were *S*-nitrosylated with 2mM GSNO (lane 3) and reduced again with 50mM DTT (lane 4). Furthermore, GSNO-treated recombinant proteins underwent the biotin switch method without ascorbate (lane 5). Proteins were separated under non-reducing conditions by SDS–PAGE and blotted onto a PVDF membrane. Biotinylated proteins were detected using an anti-biotin antibody. Data are means ±SEM of at least three replicates. *Differences from control values were significant at *P*<0.05.

In order to evaluate the potential action of different NO-derived molecules, an *in vitro* assay was carried out in the presence of ONOO^–^ using SIN-1 as the peroxynitrite donor (Chaki *et al.*, 2009; Begara-Morales *et al.*, 2014). Figure 1A depicts the inhibitory effect of ONOO^–^ on MDAR activity in a dose-dependent manner that ranges from 30% with 0.1mM SIN-1 to 67% with 1mM and 5mM SIN-1, respectively. On the other hand, Fig. 1B shows that chloroplastic GR activity was not affected by any assayed concentration of ONOO^–^. Similar behaviour was observed for cytosolic GR (results not shown).

The consistency of nitration by SIN-1 was confirmed by immunoblot analysis of the recombinant proteins using an antibody against 3-nitrotyrosine. Figure 1C and D show that the degree of nitration of both MDAR and GR isoforms increases as a function of the SIN-1 concentration.

### Effect of *S*-nitrosylation of recombinant pea MDAR and chloroplastic and cytosolic GR

In order to gain additional insight into the regulation of pea MDAR and GR proteins, the effect of increasing concentrations of GSNO, a well-known NO donor (Begara-Morales *et al.*, 2014), on the enzymatic activities was evaluated. As shown in Fig. 1E, 0.5mM and 2.0mM GSNO considerably inhibited MDAR activity by between 58% and 65%, respectively. In contrast, chloroplastic GR activity is not significantly affected (Fig. 1F). When both activities were assayed in the presence of 0.5mM and 2mM GSH to determine whether this effect was due to the release and binding of NO to the protein, GSH was found not to affect either MDAR or GR activities (Fig. 1G and H, respectively). Similar behaviour was observed for cytosolic GR (results not shown). This indicates that none of these enzymes is affected by *S*-glutathionylation. Furthermore, to show that GSNO treatment of recombinant MDAR and GR isoforms causes *S*-nitrosylation, the biotin switch assay method (Begara-Morales *et al.*, 2014) was specifically used to detect *S*-nitrosylated proteins. Figure 1I and J shows that MDAR and chloroplastic and cytosolic GR are *S*-nitrosylated after treatment with 2mM GSNO (lane 3), whereas treatment with GSH does not produce any signal in the biotin switch assay (lane 2). As expected, *S*-nitrosylation of these proteins is reversible and can be down-regulated by adding a reducing agent such as DTT to the *S*-nitrosylated proteins (lane 4) or in the absence of ascorbate (lane 5) which is used as an SNO-specific reducing agent, further demonstrating *S*-nitrosylation.

### Mapping tyrosine nitration sites and spectral characterization of nitrated pea peroxisomal MDAR

**Fig. 2.**
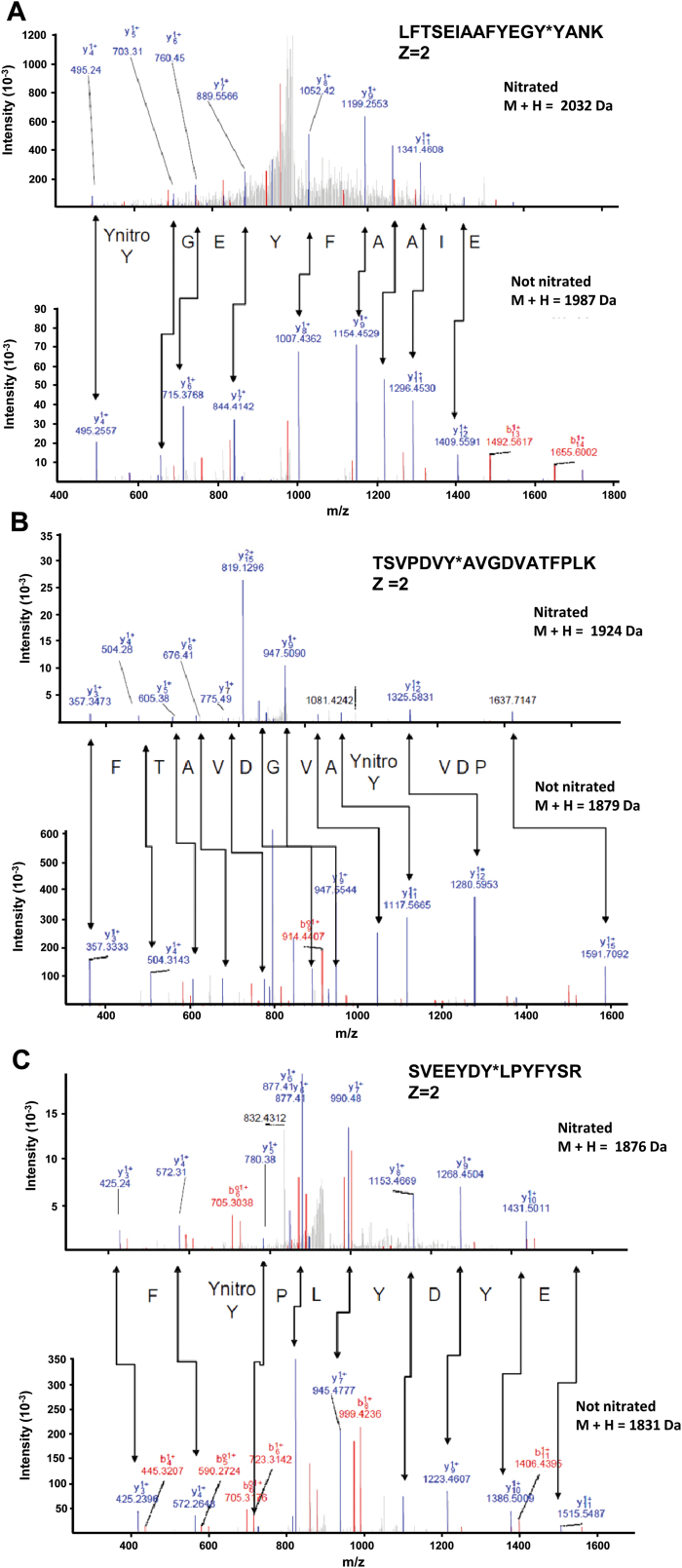
Comparison of the nitrated (top) and unmodified (bottom) MS/MS spectra of the peptides identified from the pea peroxisomal MDAR in the corresponding panels: (A) LFTSEIAAFYEGY*YANK, (B) TSVPDVY*AVGDV ATFPLK, and (C) SVEEYDY*LPYFYSR. Peptide fragment ions are indicated by ‘b’ if the charge is retained on the N-terminus and by ‘y’ if the charge is maintained on the C-terminus. The subscript indicates the number of amino acid residues in the fragment studied from either the N-terminus or the C-terminus. The superscript indicates the charge (1^+^ or 2^+^) of the backbone fragmentation. (This figure is available in colour at *JXB* online.)

Given that GR is not affected by any of the PTMs mediated for NO-derived molecules, further study was conducted on MDAR. With the aim of identifying which of the 22 tyrosines present in the pea MDAR is(are) target(s) of this PTM, peroxynitrite-treated recombinant MDAR was subjected to trypsin digestion followed by MALDI-TOF/TOF mass spectrometry examination. Table 1 shows the list of peptides scanned and those identified by LC-MS/MS. Among the peptides identified, only three contained a nitrated tyrosine. Figure 2 shows the comparison of the nitrated (top) and unmodified (bottom) MS/MS spectra of these identified peptides from the pea MDAR. The nitrated peptide LFTSEIAAFYEGY*YANK (Z=2) has a total of 17 amino acids and a mass of 2032Da (1987Da plus 45Da) which is compatible with the acquisition of a nitro group in Tyr213 (Fig. 2A). The nitrated peptide TSVPDVY*AVGDVATFPLK (Z=2) has a total of 18 amino acids and a mass of 1924Da (1879Da plus 45Da) which is also compatible with the acquisition of a nitro group in Tyr292 (Fig. 2B). The nitrated peptide SVEEYDYLPY*FYSR (Z=2) has a total of 14 amino acids and a mass of 1876Da (1831Da plus 45Da) which is also compatible with the acquisition of a nitro group in Tyr345 (Fig. 2C).

### Modelling of pea MDAR and identification of potential residues affected by peroxynitrite and GSNO

The low identity of pea MDAR with the structures available from the PDB made homology modelling a difficult task. The best co-ordinates were obtained from Geno3D server (Combet *et al.*, 2002) using as template the PDB entries 1q1w, 1q1r (26.8% identity), and 3fg2 (30.1% identity) (Sevrioukova *et al.*, 2004; Xu *et al.*, 2009). The model was refined to –18225.1 kcal mol^–1^ and comprises from residue 6 to residue 433 (98.8% coverage). The analysis of the quality of the model yields an Errat overall quality factor of 88.496, 81.82% of the residues with averaged 3D-1D score larger than 0.2, and 64.6% at the most favoured regions in the Ramachandran plot, 1.9% (i.e. seven residues) being at disallowed regions.

**Fig. 3:**
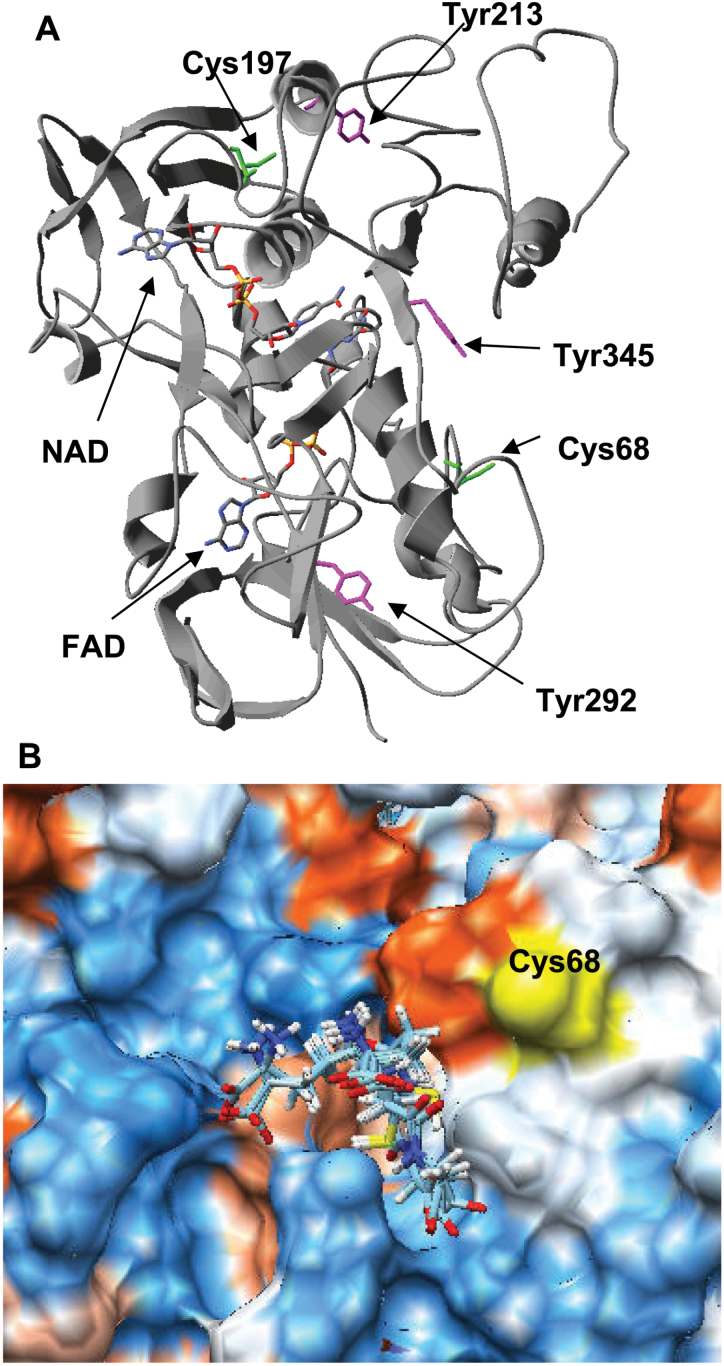
(A) Location of the tyrosine residues and cysteine residues susceptible to being responsible for the modulation of the enzymatic activity of pea MDAR by peroxynitrite and GSNO. (B) GSH binding site close to Cys68 located by blind docking. (This figure is available in colour at *JXB* online.)

The location on the model of the pea MDAR of the three tyrosine residues identified as nitrated by LC-MS/MS and the two cysteine residues that are potential targets for GSNO did not provide any conclusive information regarding the modulation of the enzyme by tyrosine nitration and/or for *S*-nitrosylation (Fig. 3A). However, since MDAR plays an important role in homeostasis, it is reasonable to assume that its regulation has been preserved through evolution. On the other hand, the fact that the modulation takes place via the modification of only a few residues points to a particular reactivity of those residues. Bearing in mind both ideas, residues identified as potential targets of the PTM were further analysed. The results are summarized in Supplementary Table S1 at *JXB* online and reveal that Tyr292 is absolutely preserved and shows the lowest estimated p*K*a, Tyr213 is prone to phosphorylation, and Tyr345 is the most accessible. Cys197 is buried and less preserved that Cys68. Molecular docking with GSNO failed due to problems with the parameterization of the S–O bond, but when the calculations were carried out with GSH an area close to Cys68 was spotted where GSH fits with an estimated *K*_d_ of 6.5 μM (Fig. 3B).

### Effect of SIN-1 on recombinant mutant pea MDAR (Tyr345Phe) obtained by site-directed mutagenesis analysis

**Fig. 4.**
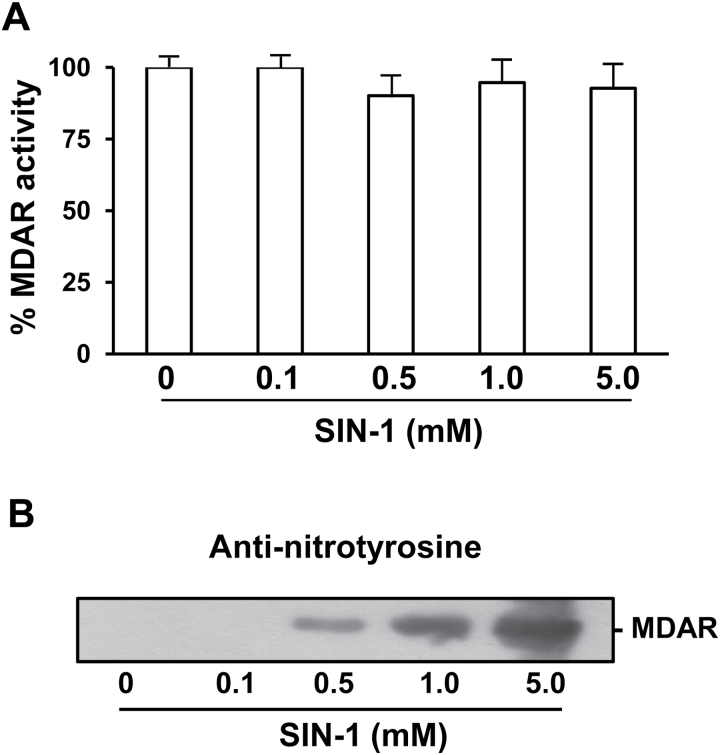
Effect of SIN-1 (peroxynitrite donor) on the recombinant mutant pea MDAR I (Tyr345Phe). (A) Effect of SIN-1 on recombinant MDAR activity. (B) Representative immunoblot showing the grade of tyrosine nitration of recombinant mutant pea MDAR and detected with an antibody against 3-nitrotyrosine (dilution 1:2500). Recombinant mutant pea MDAR I (Y345F) protein was incubated at different concentrations of SIN-1 at 37 ºC for 1h. Data are means ±SEM of at least three replicates.

Of the nitrated tyrosines, *in silico* analysis outputs Tyr345 as the most likely candidate responsible for the observed inhibition by ONOO^–^ since this residue is located at the active site and closely interacts with the cofactor. To corroborate this hypothesis, the Tyr345 residue in MDAR was replaced with phenylalanine by site-directed mutagenesis to obtain mutant MDAR Tyr345Phe. The overexpression and purification of the mutant were obtained as the recombinant wild type which yielded a functional enzyme resistant to inhibition by 0.5mM and 5.0mM ONOO^–^ (Fig. 4A). Additionally, the nitration by SIN-1 of the mutant MDAR Tyr345Phe was confirmed by immunoblot analysis using an antibody against 3-nitrotyrosine (Fig. 4B) which shows that the degree of nitration increases as a function of the SIN-1 concentration. This allows the confirmation that the nitration of Tyr345 is the residue responsible for the inhibition of MDAR activity.

### Analysis of protein and gene expression of MDAR under salt-induced oxidative stress

**Fig. 5.**
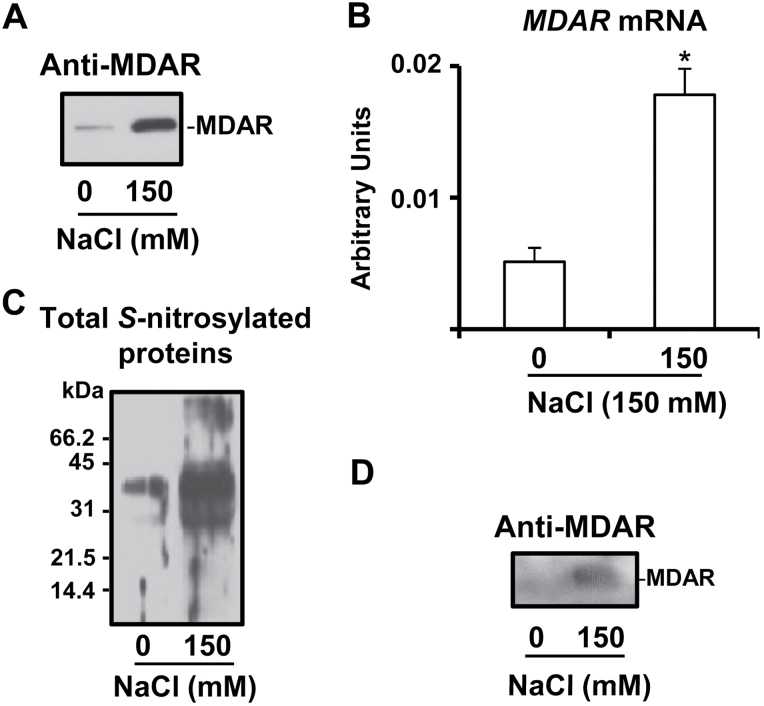
Protein and gene expression of MDAR and analysis of *S*-nitrosylated MDAR in leaves of pea plants under salinity (150mM NaCl) stress conditions. (A) Immunoblotting analysis of MDAR protein expression using an antibody against cucumber MDAR (dilution 1:3000). A 10 μg aliquot of protein was used per lane. (B) Real-time quantitative RT–PCR transcript analysis (arbitrary units) of the *MDAR* gene. Data are means ±SEM of at least four independent RNA samples. *Differences from control values were significant at *P*>0.05. (C) Detection of total *S*-nitrosylated proteins separated under non-reducing conditions by 12% SDS–PAGE and blotted onto a PVDF membrane. Biotinylated proteins were detected using anti-biotin antibodies as described in the Materials and methods. (D) Immunoblot of total *S*-nitrosylated proteins probed with an antibody against cucumber MDAR (dilution 1:3000). A 5 μg aliquot of protein was used per lane.

In order to gain additional insight into the physiological relevance of MDAR under an oxidative stress situation, it was analysed in leaves of pea plants grown in the presence of 150mM NaCl as was previously reported (Begara-Morales *et al.*, 2014). Figure 5A shows by immunoblot analysis the MDAR protein expression which was found to increase clearly under salinity conditions. *MDAR* gene expression was also analysed and a similar behaviour to the protein expression was observed, with an increase of 3.5-fold under salt stress (Fig. 5B).

### Purification of total *S*-nitrosylated proteins under salinity stress and detection of S-nitrosylated MDAR

To evaluate if MDAR under salinity stress conditions undergoes a process of *S*-nitrosylation, total *S*-nitrosylated proteins were purified from leaves of pea plants grown under control and salinity stress conditions and then the presence of MDAR protein among these S-nitrosylated protein was evaluated by immunoblotting. Figure 5D depicts the electrophoretic analysis of total *S*-nitrosylated proteins. Thus, under salinity stress, the pattern of *S*-nitrosylated proteins showed an increase in the number and in the intensity of some specific bands. Figure 5D shows the immunoblot analysis of the total *S*-nitrosylated proteins probed with an antibody against cucumber MDAR where an increase under salinity stress was also observed. Taken together, the results indicate that MDAR is *S*-nitrosylated *in vivo* and this process is increased under salinity conditions, which supports the data observed in *in vitro* conditions (Fig. 1I).

## Discussion

PTMs such as nitration and *S*-nitrosylation mediated by NO-derived molecules are now considered to be crucial elements in the fine-tuned regulation of the function of their protein targets. In higher plants, proteomic analyses of nitration/*S*-nitrosylation have shown that a certain number of proteins are targets of PTMs mediated by NO-derived molecules (Lindermayr *et al.*, 2005; Chaki *et al*., 2009; Álvarez *et al.* 2011; Lozano-Juste *et al.*, 2011; Tanou *et al.*, 2012; Begara-Morales *et al.*, 2013*a*, *b*, 2014; Corpas *et al.*, 2007, 2015). However, information on the specific impact of NO-PTMs on key proteins involved in antioxidative systems and the consequences for their functionality and protein structure is scarce (Yu *et al*., 2014).

Very recently, *in vitro* assays of several recombinant *Arabidopsis* superoxide dismutases (MnSOD1, FeSOD3, and CuZnSOD3) have shown that these SOD activities were not altered upon GSNO treatment but were inhibited to different degrees by ONOO^–^ (Holzmeister *et al.*, 2015). In this context, the ascorbate–glutathione pathway is one of the key antioxidant systems involved in the regulation of H_2_O_2_ levels in plant development and under abiotic stress conditions. In this respect, it has very recently been demonstrated that pea cytosolic APX, a key enzyme system in the antioxidant ascorbate–glutathione cycle, presents dual regulation by both tyrosine nitration and *S*-nitrosylation (Begara-Morales *et al.*, 2014). Furthermore, several proteomic studies have identified GR and MDAR, other important components in the ascorbate–glutathione cycle, as targets of tyrosine nitration/*S*-nitrosylation processes (Chaki *et al.*, 2009; Lin *et al.*, 2012; Tanou *et al.*, 2012). To elucidate the molecular mechanism and physiological relevance of these NO-PTMs to GR and MDAR functionality and their effect on the potential operation of the cycle, the present study analyses the regulation of MDAR and GR activities at the molecular level by using NO-derived molecules such as peroxynitrite and *S*-nitrosoglutathione that have the capacity to mediate tyrosine nitration and *S*-nitrosylation, respectively.

GR is an important enzyme in the antioxidative defense system that converts oxidized glutathione (GSSG) to reduced glutathione (GSH) using NADPH as cofactor. This reaction enables the GSH/GSSG ratio to be maintained at a high level, which is very important given that GSH is considered to be the most abundant soluble antioxidant in plants. This enzyme system has different isoenzymes located in a diverse range of cell compartments (Edwards *et al.*, 1990; Romero-Puertas *et al.*, 2006; Wu *et al.*, 2013) and plays an important physiological role in maintaining and regenerating GSH in response to biotic and abiotic stresses in plants (Creissen *et al.*, 1992; Foyer and Noctor, 2011; Leterrier *et al.*, 2012; Gill *et al.*, 2013; Signorelli *et al.*, 2013). Under the present experimental conditions, the activities of either isoform tested was unaffected by any NO-PTM assayed and mediated by ONOO^–^ and GSNO, suggesting that this could be a mechanism for maintaining GSH regeneration in order to sustain antioxidant capacity of the ascorbate–glutathione cycle against nitro-oxidative cell conditions. Moreover, it must be pointed out that, in the case of tyrosine nitration mediated by peroxynitrite, this behaviour is unusual as, to the authors’ knowledge, the pea GR is the first case of a nitrated protein found to be unaffected by this NO-PTM in higher plants as, up to now, most analyses have shown that nitration causes loss of function in all proteins identified in higher plants (Astier and Lindermayr, 2012; Corpas *et al.*, 2013*b*).

Given that pea GR was unaffected by NO-PTMs either for peroxynitrite or for GSNO, this study was focused on MDAR which is the enzyme in the ascorbate–glutathione cycle involved in the regeneration of reduced ascorbate. The pea (*P. sativum*) plant has only one gene encoding MDAR (Murthy and Zilinskas, 1994) whose corresponding protein has been immunolocalized in the different subcellular compartments including chloroplasts, peroxisomes, mitochondria, and the cytosol (Leterrier *et al.*, 2005). As part of the ascorbate–glutathione cycle, MDAR also plays an important role under environmental stresses in which nitro-oxidative stress could be a significant component. In pea, although MDAR activity increased under high light intensity and cadmium, it was reduced by the herbicide 2,4-D (Leterrier *et al.*, 2005). However, during the natural senescence of pea leaves, a simultaneous decrease in MDAR and APX activities has been reported (Jiménez *et al.*, 1998). In tomato, MDAR activity is also increased by salinity (Mittova *et al.*, 2003) and high light intensity (Gechev *et al.*, 2003), in rice by low temperature (Oidaira *et al.*, 2000), and in *Arabidopsis* by UV-B radiation (Kubo *et al.*, 1999). However, in *Arabidopsis*, stresses such as high temperature (30 ºC), enhanced light intensity (200 μE m^–2^ s^–1^), water deficiency (water deprivation for 2 d), and low temperature (5 ºC) did not affect MDAR activity (Kubo *et al.*, 1999).

As mentioned above, several proteomic studies have identified MDAR as a potential target for both *S*-nitrosylation and nitration (Lin *et al.*, 2012; Tanou *et al.*, 2012). However, the specific effects of these NO-PTMs on MDAR functions are not known. Thus, the present data show that both processes cause loss of MDAR function. Three putative candidates have been identified for tyrosine nitration and two cysteine residues are present in pea MDAR, but the fact that none of them is located in a relevant position from a functional point of view makes it difficult to understand the mechanism of inhibition and whether all of them are equally relevant. However since MDAR plays an important role in homeostasis and only a few residues are modified, it is reasonable to assume that those residues have been preserved through evolution and that they show a particular reactivity (Sano *et al.*, 2004). For the particular case of tyrosine nitration, it is accepted that it proceeds though a radical mechanism where tyrosine cannot react directly with peroxynitrite but rather with carbon dioxide or a metal centre to yield a secondary oxidizing species that reacts with tyrosine to form the tyrosyl radical (Alvarez and Radi, 2003). Thus, from both evolutionary and chemical points of view, Tyr292 should be the strongest candidate for nitration, Tyr345 the weakest, and Tyr213 may be discarded and considered as a good candidate to undergo phophorylation. However, an analysis of the interaction network of the protein with its cofactors shows that only Tyr345 is involved (Table 2). Thus, the second interaction sphere of Tyr345 includes residues that directly interact with the atoms N3 (His313) and N10 (Glu45 and Asp296) from FAD (Table 2). Interestingly the prediction of the p*K*a values output by PropKa 3.1 for the ionizable groups present in FAD and NAD included in the model of pea MDAR reveals that atoms N3 and N10 from FAD show extreme anomalous values, the former being –1.72 (expected 5) and the latter 4.35 (expected 10). This analysis led to the hypothesis that nitration of Tyr345 should influence the functionality of MDAR. This hypothesis was confirmed by site-directed mutagenesis and, as expected, the functionality of the mutant MDAR Tyr345Phe was not affected by 5.0mM ONOO^–^.

**Table 2.** Analysis of the first and second interaction spheres (in italics) of the three tyrosine residues identified as nitrated The contribution of hydrogen bonding (involving side chains and backbone), coulombic interactions, and desolvation effects (regular, which is calculated according to Coulomb’s law, and RE, which includes all interactions between the ionizable residue and the remaining protein, apart from the Coulomb energy, that affects the deprotonation energy of the residue). Residues of the second interaction sphere that interact with any of the cofactors are shown in bold.

Residue	p*K*a	Buried (%)	Desolvation effects		Hydrogen bond		Coulombic interaction	Atoms cofactor
			Regular	RE	Side chain	Backbone		
Tyr345 G	11.99	56 %	0.90 438	0.00 0	0.21 Asp315 G		–0.02 Arg318 0.90 Asp315	
* Asp315*	*2.63*	97	*3.82 554*	*1.17 0*	*–1.60 His313 G* *–0.85 Lys319 G* *–0.20 Tyr342 G* *–0.21 Tyr345 G*	*–0.08 Tyr345 G*	*–0.10 Arg318* *–0.32 His316* *–1.13 His313* *–1.66 Lys319*	=>N3 FAD
* Arg318*	11.00	92	*–1.93 540*	*0.00 0*			*0.21 Glu45* *0.04 Asp296* *0.10 Asp315* *0.02 Tyr345*	=>N10 FAD =>N10 FAD
Tyr292	9.95	38	1.12 387	0.00 0	–0.77 Lys285 G	0.00 Xxx0 X	0.08 Asp281 G 0.10 Asp290 –0.01 Lys102 –0.58 Lys285	
* Asp281*	3.91	32	*1.47 370*	*0.13 0*		*–0.18 Asp281 G* *–0.19 Lys285 G*	*-0.08 Lys279* *0.02 Glu 339*	
* Asp290*	*3.07*	3	*0.53 290*	*0.02 0*	*–0.85 Lys102 G*	*–0.02 Asp290 G*	*–0.03 Lys 285* *–0.38 Lys102*	
* Lys102*	11.07	0	*–0.67 268*	*0.00 0*	*0.85 Asp290 G*		*0.01 Tyr292 G* *0.38 Asp 290*	
* Lys285*	*10.17*	45	*–2.04 406*	*0.00 0*	*0.77 Tyr 292 G*		*0.34 Asp281 GX* *0.03 Asp290 G* *0.58 Tyr292*	
Tyr213	11.13	23	0.86 347	0.00 0			0.27 Asp352	
*Asp352*	*3.50*	5	*0.42 295*	*0.01 0*		*-0.52 Asp352 G* *–0.09 Leu353 G*	*–0.02 Lys217* *–0.10 Arg349*	

The enzymatic activity of MDAR is also regulated by GSNO, and a link between MDAR and NO metabolism throughout iron metabolism has been reported. Specifically, plant oxyhaemoglobin (Hb) can act as an NO scavenger with the concomitant production of nitrate and Fe^3+^-Hb which is reduced to Fe^2+^-Hb in the presence of ascorbate and NADH, and MDAR activity is a key element in this process because it facilitates the regeneration of ascorbate (Igamberdiev *et al.*, 2006; Wang and Hargrove, 2013). The sequence of MDAR comprises two cysteine residues and, according to Sano *et al.* (1995), one cysteine is more reactive towards DNTBA and it seems to participate in the reduction of the enzyme by NADH. As depicted in Supplementary Table S1 at *JXB* online, Cys68 seems to be the best candidate for *S*-nitrosylation because it is better preserved and more accessible. The identification was approached taking into account that *S*-nitrosylation by GSNO is a transnitrosylation reaction, an affinity between GSNO and the target proteins is expected, and hypothesizing that it may be detected in docking experiments. In fact it has been demonstrated that such an affinity is strong enough to be exploited to isolate targets proteins (Begara-Morales *et al.*, 2013*a*). Blind docking experiments with GSH found a region close to Cys68 where GSH fits with an estimated *K*_d_ of 6.5 μM (Fig. 3B). This result is significant because amino acid sequence alignment reveals that Cys68 is equivalent to Cys117 of chloroplastic MDAR that has been reported to be involved in the activity and structural stability of chloroplastic MDAR (Li *et al.*, 2010)

Additionally, both ONOO^–^ and GSNO showed concentration-dependent inhibitory effects on MDAR activity, which suggests that these NO-derived molecules can have a fine-tuned regulatory effect depending on their cellular production and physiological and stress conditions. It is well known that peroxisomes have a remarkable oxidative metabolism. However, it must also be pointed out that peroxisomes are subcellular compartments where the presence of l-arginine-dependent nitric oxide synthase activity (Barroso *et al.*, 1999), NO generation, and other NO-related species including ONOO^–^ and GSNO has been demonstrated (Barroso *et al.*, 2013*a*; Corpas and Barroso, 2013). Therefore, peroxisomal MDAR could be modulated through loss of function when these molecules are overproduced under adverse conditions and could consequently contribute to a nitro-oxidative stress. In order to assess the *in vivo* relevance of the present study, salt stress (150mM NaCl) was applied to plants as an inducer of nitro-oxidative stress (Begara-Morales *et al.*, 2014). It was found that MDAR expression (mRNA, protein, and activity) increased, which may reflect a mechanism to compensate the inhibitory effect of *S*-nitrosylation and nitration upon the enzyme in salt-stressed pea leaves. To date, other peroxisomal enzymes such as catalase (Clark *et al.*, 2000) and NADH-dependent hydroxypyruvate reductase (Corpas *et al.*, 2013*a*) have been demonstrated to be targets of NO, which confirms the importance of NO in the peroxisomal metabolism.

**Fig. 6.**
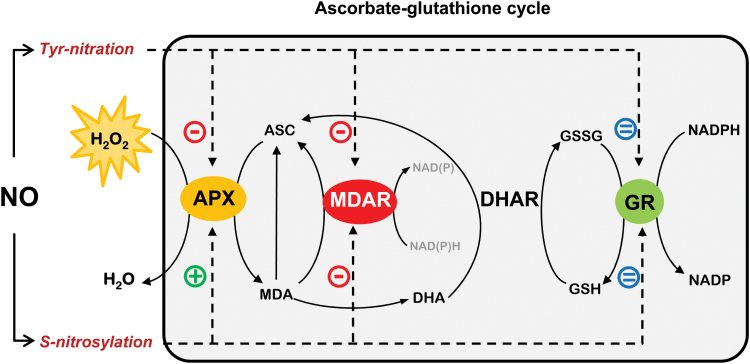
Regulation of the ascorbate–glutathione cycle by nitric oxide (NO). NO modulates the ascorbate–glutathione cycle throughout post-translational modifications (PTMs) as tyrosine nitration and *S*-nitrosylation of APX and MDAR proteins. MDAR activity is reduced after both modifications, with APX activity also being reduced by tyrosine nitration. Under nitro-oxidative stress conditions, these modifications could compromise the antioxidant capacity of the cycle. However, APX activity is enhanced by *S*-nitrosylation while GR activity is not significantly affected by these NO-related PTMs. This behaviour suggests that APX and GR try to detoxify hydrogen peroxide and maintain regeneration of GSH, respectively, and consequently the cellular redox state to maintain the antioxidant resistance of the ascorbate–glutathione cycle against nitro-oxidative cell conditions. (This figure is available in colour at *JXB* online.)

In summary, the present results provide new insights into the molecular mechanism involved in regulating MDAR and GR through PTMs mediated by NO-derived molecules and confirm the close involvement of NO and ROS metabolism in the antioxidant defence against nitro-oxidative stress situations in plants. These data, together with previous findings on the dual regulation of APX by *S*-nitrosylation/nitration (Begara-Morales *et al.*, 2014), are summarized in Fig. 6. It shows the modulation of the antioxidative response of key enzymes in the ascorbate–glutathione cycle by NO-PTMs, where MDAR was deactivated by nitration and *S*-nitrosylation, which could compromise the cycle’s antioxidant capacity. However, GR was not affected by any of these NO-PTMs in an attempt to maintain the levels of GSH and the cellular redox state, suggesting that this could be a crucial mechanism to sustain the antioxidant capacity of the ascorbate–glutathione cycle against nitro-oxidative cell conditions.

## Supplementary data

Supplementary data are available at *JXB* online.

Figure S1. SDS–PAGE analysis of the purification of the recombinant proteins.

Figure S2. Multiple alignment of the deduced amino acid sequences of MDAR in different plant species.

Table S1. Characterization in terms of evolutionary conservation, likelihood of tyrosine phosphorylation, solvent-accessible surface area, and estimated p*K*a of the three tyrosine and two cysteine residues susceptible to being responsible for the modulation of the enzymatic activity of pea MDAR by peroxynitrite and GSNO.

Supplementary Data

## Abbreviations:

NO: nitric oxide
PTM: post-translational modification
ROS: reactive oxygen species
RNS: reactive nitrogen species.
