# Supplementary material for: Differential molecular response of monodehydroascorbate reductase and glutathione reductase by nitration and S-nitrosylation
Source: J Exp Bot. 2015 Jun 25;66(19):5983–96. doi: 10.1093/jxb/erv306 (PMC4566986; doi:10.1093/jxb/erv306)
Supplement: Supplementary Data [file supp_66_19_5983__index.html]

Differential molecular response of monodehydroascorbate reductase and glutathione reductase by nitration and S-nitrosylation — Differential molecular response of monodehydroascorbate reductase and glutathione reductase by nitration and S-nitrosylation — Supplementary Data 

# Differential molecular response of monodehydroascorbate reductase and glutathione reductase by nitration and *S*-nitrosylation

## Supplementary Data

Data files

- Supplementary Data - Supplementary Data
