# Supplementary material for: Differential molecular response of monodehydroascorbate reductase and glutathione reductase by nitration and S-nitrosylation
Source: J Exp Bot. 2015 Jun 25;66(19):5983–96. doi: 10.1093/jxb/erv306 (PMC4566986; doi:10.1093/jxb/erv306)
Supplement: Supplementary Data [file supp_erv306_jexbot147389_file001.pdf]

Title: Differential regulation of monodehydroascorbate reductase (MDAR) and glutathione reductase (GR) by nitration and *S*-nitrosylation

Begara-Morales et al.

Supplemental Table 1. Characterization in terms of evolutionary conservation (rho score and residue variation in the column of the multiple alignment file), likelihood of tyrosine phosphorylation (phosphorylation score), solvent-accessible surface area (ASA) and estimated pKa of the 3 tyrosine (Y) and 2 cys (C) residues susceptible of being responsible for the modulation of the enzymatic activity of pea MDAR by peroxynitrite and GSNO

**Residue**	**Rho**	**Variability**	**NetPhos score**	**ASA(Å^2)**	**pKa**
Tyr213	3.51	YF	0.564	71	11.13
Tyr292	1.0	Y	0.402	32	9.95
Tyr345	7.14	FY.C	0.040	104	11.99
Cys68	2.58	CA		61	9.09
Cys197	5.04	LFCI		5	13.17

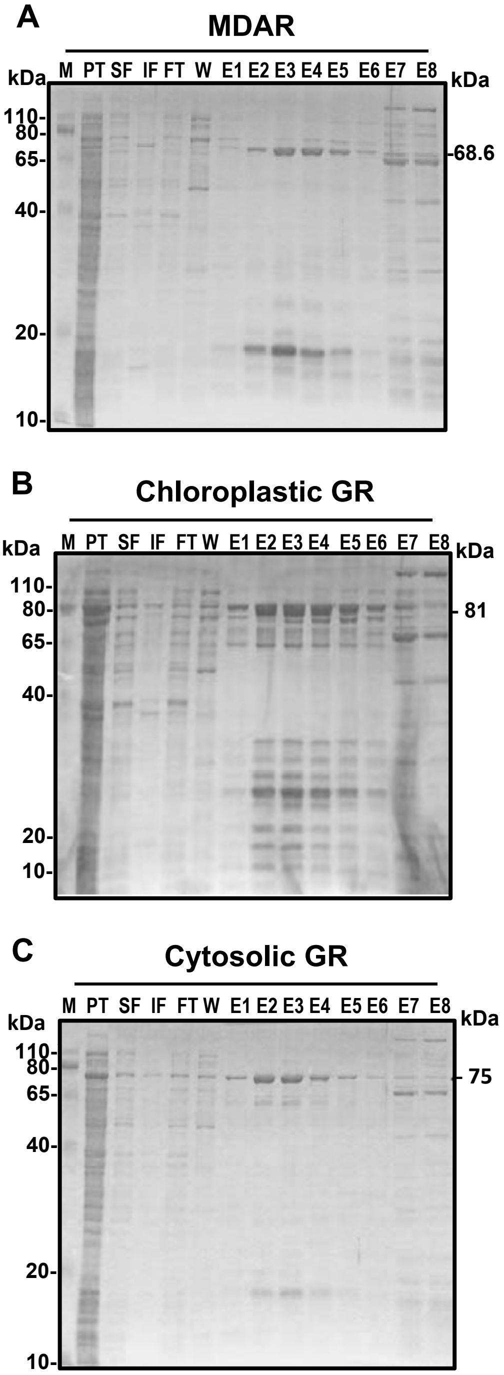

Supplemental Figure 1. SDS-PAGE analysis of the purification of the recombinant proteins. (A) Peroxisomal monodehydroascorbate reductase (MDAR). **(B)** Chloroplastic glutathione reductase. **(C)** Cytosolic glutathione reductase. The gel was stained with Coomasie blue. M, molecular markers; PT, total protein; SF, soluble fraction; IF, insoluble fraction; FT, flow-through; W, wash; E1-E8, elution fractions.

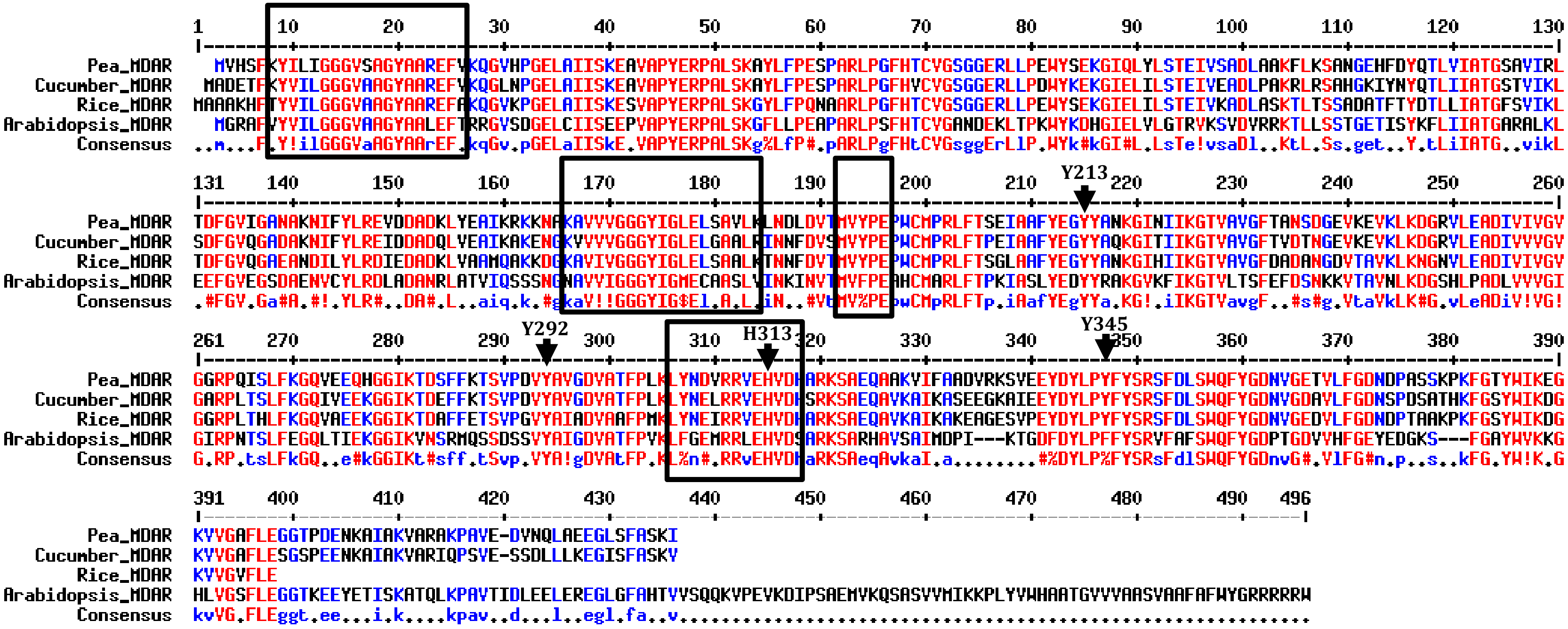

Supplemental Figure 2. Multiple alignment of the deduced amino acid sequences of MDAR in different plant species. Nucleotide binding zones are highlighted within a box. Arrows indicate tyrosines targets of nitration (Y213, Y292 and Y345) and Histidine 313. Alignment was carried out using Multalin (http://multalin.toulouse.inra.fr/multalin/). Pea_MDAR: MDAR from *Pisum sativum* (AAV11490.1); Cucumber_MDAR: MDAR from *Cucumbis sativa* (BAA05408.1); Rice_MDAR: MDAR from *Oryza sativa* (BAA77282.1); Arabidopsis_MDAR: MDAR from *Arabidopsis thaliana* (AEE77367.1).
